# Contribution of circulatory cells to asthma exacerbations and lung tissue-resident CD4 T cell memory

**DOI:** 10.3389/fimmu.2022.951361

**Published:** 2022-07-22

**Authors:** Gurupreet S. Sethi, Donald Gracias, Michael Croft

**Affiliations:** ^1^ Center for Autoimmunity and Inflammation, La Jolla Institute for Immunology, La Jolla, CA, United States; ^2^ Department of Medicine, University of California San Diego, La Jolla, CA, United States

**Keywords:** asthma, circulatory memory T cells, tissue-resident memory T cells, Trm, FTY720

## Abstract

Tissue-resident memory CD4 T cells (Trm) are thought to be a major contributor to asthma relapse, but the role of circulatory T cells in asthma exacerbations or to maintaining the population of lung Trm cells is not fully understood. Here, we used a house dust mite allergen-based murine model of asthma relapse, and monitored the development of lung effector/Trm phenotype CD44^hi^CD62L^lo^CD69^+^ CD4 T cells. To determine the contribution of circulatory cells, mice were treated with FTY720, to block lymphocyte egress from lymph nodes. Inhibiting the primary migration of circulatory cells to the lungs mitigated the accumulation and expansion of allergen-driven Trm phenotype cells, but subsequent allergen challenges still resulted in strong lung inflammation and Trm cell accumulation. This was blocked if FTY720 was also given at the time of allergen re-exposure, showing that new circulatory cells contributed to this lung memory/effector T cell pool at times well after the initial sensitization. However, once lung-localized Trm cells developed at high frequency, circulatory cells were not required to maintain this population following allergen re-encounter, even though circulatory cells still were major contributors to the overall asthmatic lung inflammatory response. Our results suggest that strategies that target the response of circulatory memory T cells and Trm cells together might be required to strongly inhibit T cell reactivity to airborne allergens and to limit exacerbations of asthma and their reoccurrence, but the contribution of circulatory T cells might vary in long-term asthmatics possessing a large stable Trm cell population in the lungs.

## Introduction

Asthma is a chronic inflammatory disease of the lungs that affects around 350 million people worldwide. Most asthmatics are hypersensitive to many allergens, including from house dust mites (1). Allergen-activated CD4 T cells play a critical role in orchestrating acute disease features such as recruitment of eosinophils, airway inflammation, and mucus production, that ultimately leads to airway obstruction in asthma patients (2, 3). As importantly, it is believed that long-lived populations of lung-localized CD4 memory T cells, also referred to as tissue-resident memory T cells or Trm (4–7), might be responsible for persisting disease, and are the cell type that initiates periodic exacerbations of lung inflammation upon encounter with airborne allergens. From human and mouse lung studies, it has been estimated that around 90% of the initial effector CD4 T cell pool dies after allergen exposure, and a small subset of Trm cells of around 5-10% can survive in the lungs, at least as a population, for an extended period of time (8–11). Indeed, these allergen-induced memory T cells have been visualized in the mouse lungs for more than two years (12). Importantly, many Trm cells appear to reside in or near the epithelium lining of the lung mucosa potentially allowing them to react quickly to mucosal antigen (11, 13).

Central and effector memory T cells can also develop, and can expand in response to antigen reencounter in local tissue draining secondary lymphoid organs, and further can migrate into the tissues where antigen is expressed. Thus, while Trm cells might be the first line of reactivity when new antigen is available, central or effector memory T cells, collectively referred to as circulatory T cells, could contribute to acute tissue inflammation in concert with Trm cells. In the case of allergen-driven asthmatic lung inflammation, this possible role for circulatory T cells has received less attention, including whether circulating memory T cells are required to maintain the persisting Trm cell pool after it initially forms. Notably, a recent study by Rahami and coworkers, using a parabiosis model, found that both CD4 circulatory memory T cells and Trm played a role in acute inflammation driven by airway allergen (11). Specifically, lung-resident cells were sufficient to induce mucus production, airway hyper-responsiveness, and eosinophilia, whereas circulatory memory T cells promoted inflammation localized to the lung blood vessels and formed part of the total pool of effector/memory T cells in the lungs.

Thus, while strategies that could directly target Trm cells in asthmatics is likely to lead to significant benefit, other strategies that might dampen the effects of circulatory memory T cells might also be useful. In this regard, the S1P receptor modulator, FTY720, or similar molecules, that can alter T cell trafficking from lymph nodes, have been investigated in a number of model systems, and subsequently found therapeutically efficacious in reducing disease in patients with multiple sclerosis and ulcerative colitis (14, 15). Older studies in acute asthma models driven by OVA as the antigen found that FTY720 treatment could reduce lung inflammation with changes in T cell activity (16–18). However, whether inhibiting circulating cells from entering the lungs controls long-term memory T cell-induced exacerbations of lung inflammation, or affects lung Trm cell persistence has not been fully addressed.

In the present study, we used a house dust mite allergen-based murine model of relapsing asthma to understand the impact of circulatory cells to disease progression and disease maintenance by treating mice with FTY720 at various phases during and after the formation of pools of persisting Trm cells. Our results demonstrate that blocking the migration of circulatory cells during initial allergen encounters limited the formation, expansion, and activity of effector Trm phenotype cells, and prevented the long-lasting persistence of Trm cells in the lungs. In contrast, once lung Trm cells developed at high frequency, they no longer relied on circulatory cells for their expansion and for the further survival of the lung Trm pool. However, circulatory cells still contributed to the effector memory T cell population that drove exacerbations of acute inflammation. These data suggest that reagents that control allergen-induced circulatory cell migration into the lungs could be useful in asthmatics to dampen acute inflammatory activity, but their effectiveness might be dependent on the frequency of the long-lasting Trm pool that has formed and persists in allergen-experienced lungs.

## Material and methods

### Mice

Female C57BL/6 mice of 6-8 weeks old were purchased from The Jackson Laboratory (Bar Harbor, ME). Foxp3-GFP reporter mice on a BL/6 background were bred in house. All experiments were conducted in accordance with the regulations of the La Jolla Institute for Immunology Animal Care Committee (IACUC # AP00001042).

### Experimental protocols and FTY720 treatment

All mice, with the exception of the naïve group, were sensitized systemically by intraperitoneal (i.p.) injection of a mixture of 20 μg HDM extract (Greer Labs, Lenoir, NC) and 2 mg alum. After a resting period of 10 days, sensitized mice were challenged with 10 μg of HDM given i.n. for four-consecutive days (10, 11, 12, and 13, referred to as secondary allergen exposure). In some experiments, sensitized and challenged mice were rested for a further period of 4 weeks (up to day 38) before analysis, and also re-challenged with same allergen (10 μg HDM; i.n.) for another four consecutive days (40, 41, 42, and 43, referred to as tertiary allergen exposure). To assess the impact of circulatory T cells on lung responses, mice were administrated i.p. FTY720 (Fingolimod; Calbiochem, San Diego, CA; 10mg/ml in DMSO) or vehicle (DMSO) alone, daily for 6 consecutive days during the allergen encounters (secondary and/or tertiary response) starting two days before allergen. To distinguish cells in the lung tissue *vs*. lung vasculature, mice were injected i.v. with fluorophore-labelled anti-CD90.2 (2 μg in 100ul of PBS; APC eF780, clone 30-H12; Invitrogen) through the retro-orbital sinus 10 minutes before tissue harvest to label intravascular cells.

### Bronchoalveolar lavage and lung tissue analysis

Mice were euthanized at varying time points (days 14, 38, 45 and 71). Trachea were exposed, cannulated, and lungs were lavaged with chilled PBS (2% BSA) to collect Bronchoalveolar lavage fluid (BALF). Total cell counts were determined using a Hemocytometer and differential cell counts were determined with flow cytometry. For histopathological analysis, lungs were fixed in zinc formalin buffer. Paraffin-embedded sections of 5-6 μm were stained with hematoxylin and eosin (H&E) or Periodic acid–Schiff (PAS) to examine inflammatory cell infiltration and mucus production, respectively. Images were acquired on a Zeiss AxioScan Z1 slide scanner. At least five fields (400x magnification) in each lung section were examined blinded for the quantification. The inflammatory cell infiltration around the bronchioles was quantified using a 0-5 scoring system; 0 indicates no inflammatory cells; 1, a few dispersed inflammatory cells; 2, a ring of inflammatory cells one cell‐layer deep; 3, a ring of inflammatory cells two cells deep; 4, a ring of inflammatory cells three-four cells deep; and 5, a ring of inflammatory cells >four cells deep. PAS stained sections were scored from 0-4, reflecting <5-25%, 25-50%, 50-75% and >75% mucus positive epithelial cells per bronchiole, respectively.

### Flow cytometry

Lung tissue was digested using a mouse lung dissociation kit (MACS-Miltenyi Biotec) and homogenized to single cell suspensions with gentleMACS™. Dissociators (MACS-Miltenyi Biotec). Approximately 2 x10^6^ cells were processed for staining. First, single cells were incubated with anti-mouse CD16/32 (clone 93, TruStain fcX, BioLegend) for 5 min at room temperature to block Fc receptors. Next, lung cells were stained with live dead stain (Invitrogen™ LIVE/DEAD™ Fixable Blue Dead Cell Stain Kit) for 30 mins at RT to exclude dead cells, and then further stained with panel A and panel B for 30 mins at 4 degree to assess eosinophils and different subsets of memory CD4 T cells, respectively. Panel A included the following anti-mouse antibodies: from BioLegend, San Diego, CA: AF700-CD45 (clone 30-F11), BV650-CD8a (clone 53-6.7), PE-SiglecF (clone S17007L), BV510-CD11b (clone M1/70) and PerCP Cy5.5-Ly6G (clone 1A8); from Invitrogen, Carlsbad, CA: APC-TCRb (clone H57-597), PEeF610-B220 (clone RA3-6B2) and eF450-CD11c (clone N41B); and from BD bioscience, San Jose, CA: BUV395-CD4 (clone GK1.5)]. Panel B consisted of anti-mouse antibodies: from BioLegend, San Diego, CA: AF700-CD45 (clone 30-F11), BV650-CD8a (clone 53-6.7), BV570-CD62L (clone MEL-14), BV421-Foxp3 (clone MF-14) and PEcy7-CD69 (clone H1.2F3); from Invitrogen, Carlsbad, CA: APC-TCRβ (clone H57-597), PEeF610-B220 (clone RA3-6B2) and eF450-CD11c (clone N41B); and from BD bioscience, San Jose, CA: BUV395-CD4 (clone GK1.5) and BUV737-CD44 (clone IM7)]. Either Foxp3-GFP mice were used, or intracellular staining for Foxp3 was performed using eBioscience™ Foxp3/Transcription Factor Staining Buffer kit, to exclude Treg. Similarly, BALF cells were stained with the above-mentioned panel A to assess eosinophils. Stained cells were analyzed on a BD LSR Fortessa X-20 flow cytometer (BD Biosciences) and with FlowJo software (Tree Star). Total numbers of inflammatory cells were calculated using relative frequencies.

### *In vitro* analysis of T cell responsiveness

2 x10^6^ lung cells were ex vivo stimulated with HDM (10 ug/ml, Greer Labs, Lenoir, NC) and IL-2 (5ng/ml, PeproTech, Cranbury, NJ, USA) for 4 days and production of IL-5 and IL-13 measured by intracellular flow in gated CD4^+^Foxp3^-^ T cells using the following anti-mouse antibodies: BV605-CD45 (clone 30-F11, BioLegend), BV711-TCRB (clone H57-597, BioLegend), APC-CD4 (clone GK1.5, BioLegend), PE-IL5 (clone TRFK5, BD bioscience) and PReF610-IL13 (clone eBio13A, Invitrogen).

### Statistical analyses

Results are presented as mean ± SEM. All statistical analyses were performed using GraphPad Prism software (GraphPad Software, La Jolla, CA, USA). To compare two experimental groups, Student’s t test followed by Mann–Whitney U test was performed, whereas for multiple group data, one-way ANOVA followed by Holm-Sidak’s multiple comparisons test was performed. Differences between the groups were considered as statistically significant with p<0.05. For individual graphs, *p< 0.05, **p< 0.01, ***p< 0.001, ****p< 0.0001.

## Results

### Periodic airway allergen exposure induces persisting memory CD4 T cells in the lung vasculature and the lung tissue

**Figure 1:**
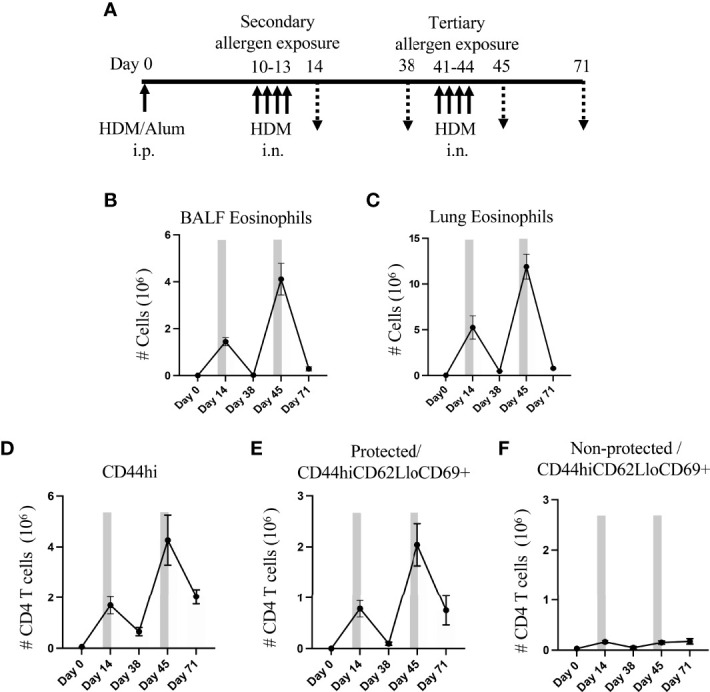
Lung Trm phenotype cells strongly expand with recurrent intranasal HDM challenges. **(A)** Protocol for sensitization and challenge with HDM. Mice were analyzed on days 14, 38, 45 and 71. **(B, C)** Number of BALF **(B)** and lung **(C)** eosinophils. D) Number of lung tissue CD44hi memory/effector CD4 T cells, and protected and non-protected CD44hiCD62LloCD69+ Trm phenotype CD4 T cells. Bars depict the allergen exposure windows. Data means ± SEM, n = 4-8 mice per group, from 2 representative experiments.

To understand the influence of circulatory cells on the development and maintenance of lung-resident memory CD4 T cells, we used an HDM allergen-based murine model of lung inflammation. We sensitized mice systemically by administering a mixture of HDM and alum intraperitoneally as in previous studies (19), and then challenged mice intranasally with the same allergen for a period of four consecutive days in an acute secondary response. This was followed by a resting period of 4 weeks that allowed the generation of HDM-reactive memory CD4 T cells, and a further intranasal challenge to elicit a tertiary response. Mice were analyzed at different time points as shown in **Figure 1A** (days 14, 38, 45 and 71) to assess CD4 memory/effector T cells in the lung for their accumulation, expansion and persistence. In our model a blocking antibody to CD3 can effectively reduce lung inflammation (unpublished data), and so as an indirect readout of the activity of the responding T cells, we assessed eosinophilia in the bronchoalveolar lavage fluid or lungs (**Figures 1B, C**), which is the primary asthmatic phenotype, and general lung inflammation by histology.

Flow cytometry analysis at day 14 showed that the number of eosinophils significantly increased in sensitized mice upon secondary allergen exposure and then decreased after a resting period of 4 weeks (day 38), almost to the level in unsensitized mice. Tertiary allergen exposure resulted in much greater eosinophilia (~3-fold higher in BALF and ~2.2-fold higher in lung; day 45 *vs*. day 14), suggesting strong development of T cell memory. This again was followed by resolution of the inflammatory eosinophil infiltrate over the next few weeks (~93% reduction in both BALF and lung tissue at day 71 *vs*. day 45), although interestingly a substantial number of eosinophils still remained in the lung tissue at this late time (**Figures 1B, C**).

To assess accumulation of allergen-induced tissue-resident memory (Trm) phenotype T cells, we enumerated CD44^hi^CD62L^lo^ CD4 T cells that expressed CD69 and were negative for Foxp3 (**Supplementary Figure 1**). To discriminate between those extravascular cells in the lung tissue versus cells in the vasculature, mice were given a short-term i.v. injection of fluorophore-labelled anti-CD90.2 antibody before lung harvesting. We refer to the CD90.2 stained cells in the lung circulation/vasculature as ‘non-protected cells’ and the unstained extravascular lung tissue-resident cells as ‘protected cells’.

Paralleling the eosinophil response, lung memory/effector (CD44^hi^) CD4 T cells accumulated over time, including protected and non-protected CD44^hi^CD62L^lo^CD69^+^ Trm phenotype cells (**Figures 1D–F**). The secondary allergen exposure (day 14) resulted in an enhanced number of non-protected Trm phenotype T cells in the lung vasculature (~5-fold above that in naive animals at day 0), whereas protected lung-localized Trm phenotype cells were dramatically increased by ~2600-fold over those in naive lungs. The total number of protected lung T cells was far in excess of the non-protected T cells (~470-fold) at this time. After the allergen challenge, only a small fraction of ~10% of the protected Trm phenotype cells were still retained in the lungs 4 weeks later (day 38), while ~30% of the non-protected lung Trm phenotype cells were still visualized during the period following allergen challenge (**Figures 1E, F**).

Upon tertiary allergen exposure (days 41-44), a significant increase in the number of total CD4 memory T cells (~6-fold, **Figure 1D**) and protected CD44^hi^CD62L^lo^CD69^+^ subset (~20-fold, **Figure 1E**) was observed in comparison to the resting number on day 38, which was 2.5-fold and 2.6-fold greater, respectively, than the numbers that accumulated in the secondary response at day 14. However, although the number of non-protected Trm phenotype cells in the lung vasculature increased with tertiary challenge (3-fold compared to day 38), their accumulation was almost identical to that seen at day 14 (**Figure 1F**).

Our results further indicated that these allergen-induced memory CD4 T cells were long-lasting as a population as many were retained over the next 4 weeks following the tertiary allergen response (day 71). Around 35% of the population of protected CD44^hi^CD62L^lo^CD69^+^ Trm phenotype cells that accumulated at day 45 were still in the lungs at day 71, which was 3.5-fold higher than were retained in the lungs after the secondary response at day 38 (**Figure 1E**). In contrast, numbers of the non-protected Trm phenotype cells found in the lung vasculature at this late time (day 71) were similar to those visualized after the secondary exposure (day 14) and the tertiary response (day 45) (**Figure 1F**). Overall, repeated HDM challenge, followed by periods without allergen exposure, led to the development of high frequencies of persisting Trm phenotype CD4 T cells both in the lung vasculature (non-protected cells) and the lung tissue (protected cells), although the predominant population was in the tissue.

### Development of lung tissue-resident memory phenotype T cells upon initial inhalation of allergen depends on egress from the lymph nodes

**Figure 2:**
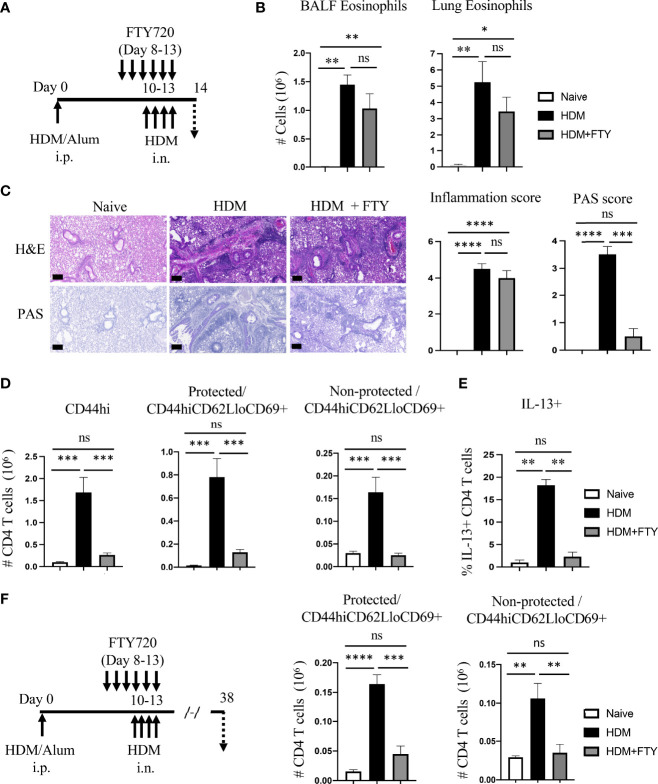
FTY720 treatment during acute HDM exposure impairs the development of lung tissue-resident memory CD4 T cells. **(A)** Experimental protocol. Analysis on day 14. **(B)** Number of BALF and lung eosinophils. **(C)** H&E and PAS staining (scale bar, 100 μm), and scoring for inflammation around the bronchioles and bronchial epithelial mucus production. **(D)** Number of lung tissue CD44hi, and protected and non-protected CD44hiCD62LloCD69+ CD4 T cells. **(E)** Percentage of IL-13 producing lung CD4 T cells. **(F)** Experimental protocol. Analysis on day 38. Number of protected and non-protected CD44hiCD62LloCD69+ CD4 T cells. Data means ± SEM, n = 4-5 mice per group, representative of 2 experiments.

The importance of circulatory cells in contributing to tissue-resident memory T cells and to lung inflammation is unclear. Also, whether preventing migration of cells from the lymph nodes to the lungs could be useful therapeutically to reduce Trm cell frequency has not been investigated thoroughly. To pursue these questions, we first treated mice with FTY720 to block lymph node egress during the acute secondary response where HDM-sensitized mice were challenged i.n. for four-consecutive days from day 10 to 13 (**Figure 2A**). Surprisingly, little effect was seen on BALF and lung eosinophilia with only a slight statistically non-significant reduction (**Figure 2B**). Histopathological analysis also showed only a moderate difference in peri-bronchial inflammation where eosinophils localize. However, the production of mucus by bronchial epithelial cells was almost completely suppressed (**Figure 2C**). Flow cytometry analysis demonstrated that FTY720 significantly reduced the accumulation of total memory CD4 T cells, including both protected and non-protected CD44^hi^CD62L^lo^CD69^+^ T cells (**Figure 2D**), although a small fraction of protected Trm phenotype T cells remained. This shows that blocking the migration of circulatory cells to the lungs mitigated the expansion of the majority of Trm phenotype cells seen when HDM was first inhaled into the lungs. Corresponding to reduced mucus production, where IL-13 is the main inducer of this phenotype, the percentage of IL-13 positive CD4 T cells in the lungs was similarly decreased in FTY720-treated mice (**Figure 2E**). However, this effect on T cells did not explain why eosinophilia was relatively normal. To gain more insight, sensitized mice were analyzed on day 8 before FTY720 treatment and allergen challenge (**Supplementary Figure 2A**). This showed that systemic allergen sensitization drove recruitment of some CD4 memory phenotype T cells into the lung tissue, and these made IL-5 but not appreciable IL-13 (**Supplementary Figures 2B–D**). Given the major role of IL-5 in the recruitment of eosinophils, this likely explained why FTY720 treatment was ineffective in substantially reducing eosinophilia even though the accumulation of the majority of memory phenotype CD4 T cells was impaired upon HDM exposure.

This data was then extended to assess the impact of FTY720 on the accumulation of a more resting population of Trm phenotype T cells that persist following the expansion and contraction of the secondary response. FTY720-treated mice were allowed to recover for a period of 4 weeks and analyzed on day 38 (**Figure 2F**). The total number of protected as well as non-protected Trm phenotype cells was significantly reduced in FTY720-treated mice, although again a small population of protected cells still accumulated (~30% of those in control animals challenged with HDM; ~3-fold above that in untreated mice) (**Figure 2F**). Overall, these results indicate the critical role of the influx of circulatory cells during initial allergen encounters in the airways for the generation of lung-localized and vasculature-localized tissue-resident memory T cell populations.

### Blocking lymph node egress during initial airway allergen exposure only partially prevents the accumulation of lung Trm phenotype CD4 T cells upon further allergen exposure

**Figure 3:**
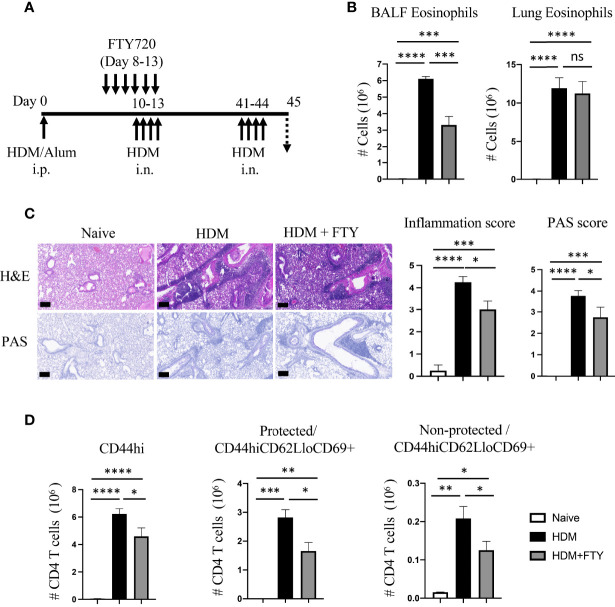
FTY720 treatment during acute secondary allergen response partially reduces re-expansion of lung Trm. **(A)** Experimental protocol. Analysis on day 45. **(B)** BALF and lung eosinophilia. **(C)** H&E and PAS staining (scale bar, 100 μm), and scoring for inflammation around the bronchioles and bronchial epithelial mucus. **(D)** Number of lung tissue CD44hi, and protected and non-protected CD44hiCD62LloCD69+ CD4 T cells. Data means ± SEM, n = 3-5 mice per group. ns, *, **, *** and **** stand fornon-significant, P < 0.05, P < 0.01, P < 0.001, and P<0.0001, respectively.

Given the substantial reduction in the generation of Trm cells on day 38 after FTY720 treatment during the secondary allergen challenge, we then asked how this affected the expansion of Trm phenotype effector cells upon re-exposure with inhaled allergen during a tertiary response. FTY720-treated and rested mice were given HDM i.n. on days 41-44 (**Figure 3A**). Interestingly, lung eosinophilia on day 45 was essentially the same as that in mice not given FTY720 although a reduction in BALF eosinophilia was observed (**Figure 3B**). H&E and PAS staining of lung tissue revealed that FTY720 treatment slightly suppressed the HDM-induced inflammatory infiltrate as well as mucus production (**Figure 3C**). In accordance, although FTY720 treatment led to a 20-30% reduction in the accumulation of total memory CD4 T cells, and a 30-40% reduction in both protected and non-protected CD44^hi^CD62L^lo^CD69^+^ populations, a substantial number of Trm phenotype cells were still observed in the lung tissue (38-fold above the number on day 38) and lung vasculature (3.5-fold above day 38) (**Figure 3D**). Overall, these results established that maximal accumulation of lung Trm phenotype cells required migration of circulatory cells during the secondary response to allergen. The latter results also could have suggested that the small number of lung Trm cells persisting after the secondary response expanded well during the subsequent allergen encounter to account for the number found in FTY720-treated animals. Alternatively, new circulatory cells contributed to the pool of T cells that accumulated in the lungs during this tertiary response.

### Blockade of circulatory cell migration during initial and repeated allergen encounters prevents the expansion and persistence of lung Trm cells

**Figure 4:**
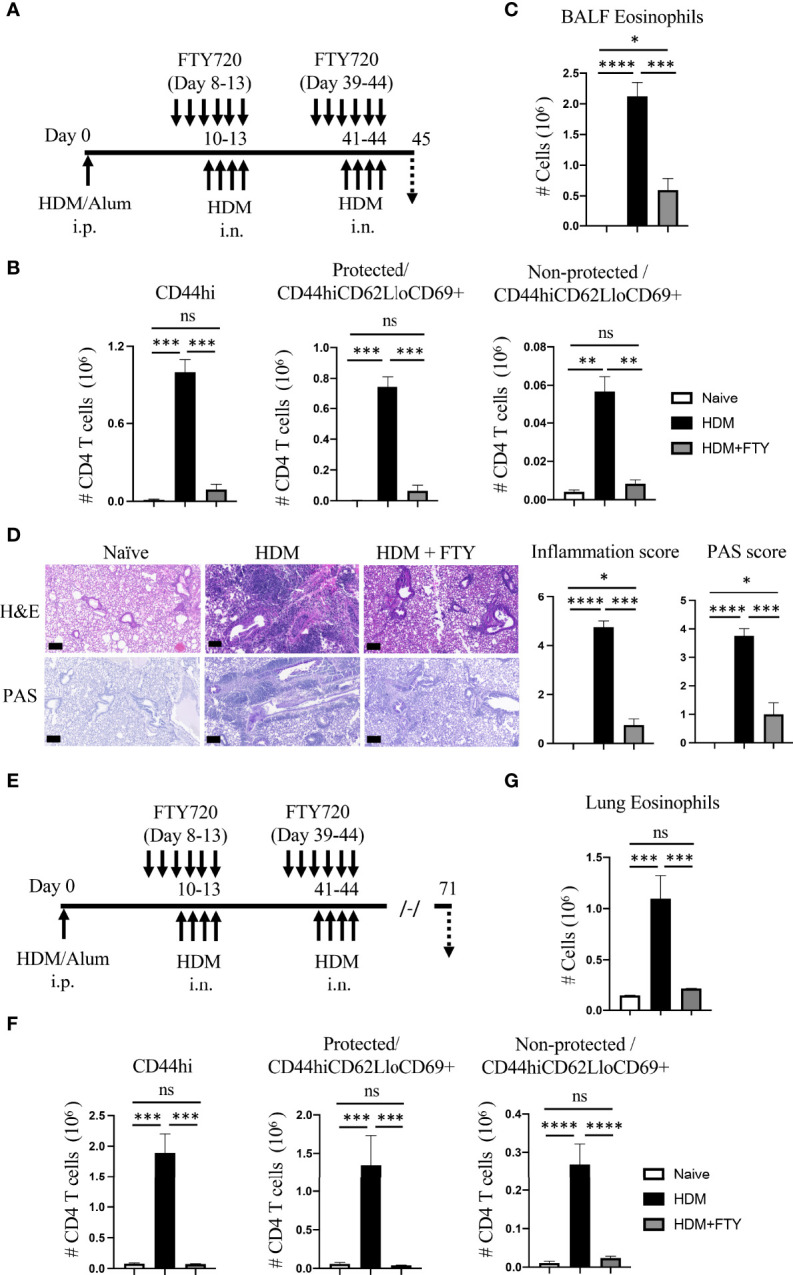
Administration of FTY720 during secondary and tertiary allergen exposures blocks the expansion and persistence of lung Trm cells. **(A)** Experimental protocol. Analysis on day 45. **(B)** Number of lung tissue CD44hi, and protected and non-protected CD44hiCD62LloCD69+ CD4 T cells. **(C)** BALF eosinophil numbers. **(D)** H&E and PAS staining (scale bar, 100 μm), and scoring for inflammation and mucus. **(E)** Experimental protocol. Analysis on day 71. **(F)** Number of lung tissue CD44hi, and protected and non-protected CD44hiCD62LloCD69+ CD4 T cells. **(G)** Lung eosinophil numbers. Data means ± SEM, n = 3-5 mice per group, representative of 2 experiments. ns, *, **, *** and **** stand fornon-significant, P < 0.05, P < 0.01, P < 0.001, and P<0.0001, respectively.

To address the latter and assess whether influx of circulatory cells during the tertiary allergen exposure contributed to the pool of lung-localized Trm phenotype cells, mice were then treated with FTY720 during both the secondary and tertiary allergen challenges (**Figure 4A**). This led to almost complete ablation of the expansion and accumulation of all memory CD4 T cell populations after the tertiary challenge at day 45, including both protected and non-protected CD44^hi^CD62L^lo^CD69^+^ Trm phenotype cells (**Figure 4B**). Correspondingly, there was strongly reduced eosinophilia and lung tissue inflammation with repeat FTY720 treatments (**Figures 4C, D**). Moreover, when mice were analyzed 4 weeks later (day 71; **Figure 4E**), serial FTY720 treatment effectively blocked the generation of persisting Trm cells in both the lung tissue (protected) and lung vasculature (non-protected) (**Figure 4F**; **Supplementary Figure 3**). In line with this, the persisting tissue eosinophilia seen at day 71 in untreated mice was absent (**Figure 4G**). Thus, if circulatory cell migration was inhibited by FTY720 treatment before the establishment of a large pool of stable tissue-resident memory T cell populations, and treatment was continued, this strongly impacted the accumulation of sufficient numbers of effector memory T cells in the lung, impaired lung inflammation, and blocked the generation of persisting Trm cells in the lungs.

### Circulatory cells are not required for the expansion and persistence of already developed lung-localized memory T cells but still contribute to inflammation

**Figure 5:**
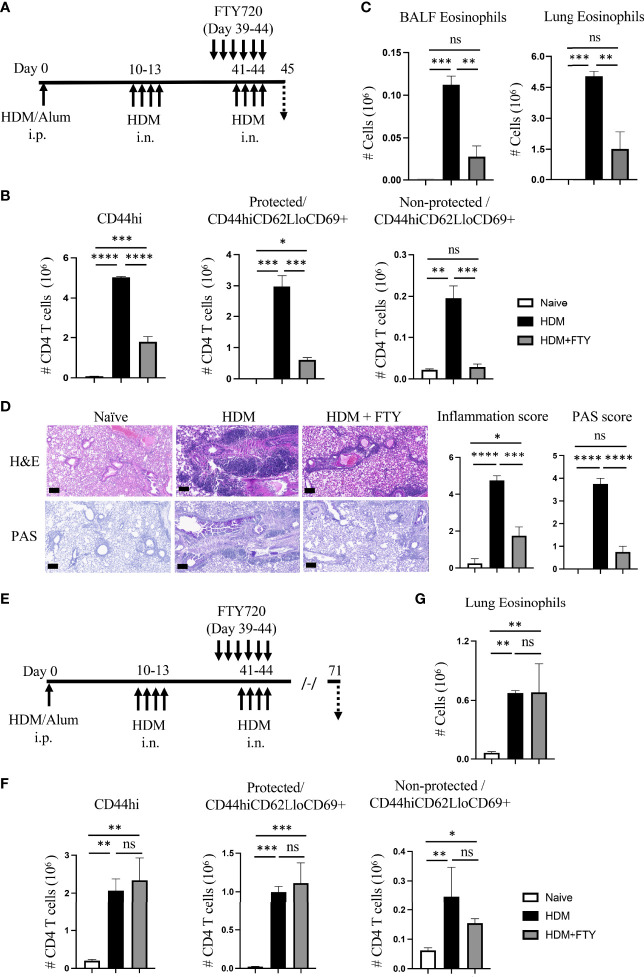
Blocking circulatory CD4 T cells with FTY720 during tertiary allergen exposure inhibits expansion of Trm and lung inflammation but does not affect the long-term persistence of Trm. **(A)** Experimental protocol. Analysis on day 45. **(B)** Number of lung tissue CD44hi, and protected and non-protected CD44hiCD62LloCD69+ CD4 T cells. **(C)** BALF and lung eosinophil numbers. **(D)** H&E and PAS staining (scale bar, 100 μm), and scoring for inflammation and mucus. **(E)** Experimental protocol. Analysis on day 71. **(F)** Number of lung tissue CD44hi, and protected and non-protected CD44hiCD62LloCD69+ CD4 T cells. **(G)** Lung eosinophil numbers. Data means ± SEM, n = 3-5 mice per group, representative of 2 experiments. ns, *, **, *** and **** stand fornon-significant, P < 0.05, P < 0.01, P < 0.001, and P<0.0001, respectively.

Lastly, we asked if migration of circulatory cells played a role in lung inflammation and the expansion and persistence of Trm phenotype populations after Trm cells had already developed in high numbers in the allergic lungs. HDM-exposed mice were only treated with FTY720 during the tertiary allergen challenge and analyzed on day 45 (**Figure 5A**). The total number of lung memory T cells, and protected as well as non-protected Trm phenotype cells was significantly decreased in FTY720 administrated mice (**Figure 5B**). Similarly, FTY720 treatment also limited the HDM-induced eosinophilia (**Figure 5C**), and substantially reduced peribronchial infiltration and mucus production (**Figure 5D**). This data demonstrates that circulatory cell egress from the lymph nodes still contributes to the effector memory T cell population that accumulates in the lungs, and to the level of inflammation, even when high numbers of Trm cells have already seeded the lungs.

Notably, however, a proportion of protected Trm phenotype cells (~0.6 x 10^6^, **Figure 5B**) were still present in the lungs in FTY720-treated animals at higher levels (~4-fold increase) than seen before the tertiary challenge (~0.16 x 10^6^, HDM group, **Figure 2F**). This corresponded approximately to the remaining eosinophilia induced in the FTY720-treated animals (**Figure 5C**). Moreover, 4 weeks later (day 71, **Figure 5E**), the total number of memory CD4 T cells remaining in the lungs was not different in the FTY720-treated animals, with the protected lung-localized Trm cells maintained at a level equivalent to that in mice not administered FTY720 (**Figure 5F**). Interestingly, this was also comparable to the number of protected Trm phenotype cells observed at day 45 in FTY720-treated mice (compare to **Figure 5B**). Lastly, in line with this Trm cell population being unaffected, the persisting tissue eosinophilia seen at day 71 in untreated mice was present in FTY720-treated mice (**Figure 5G**). Therefore, once allergen-induced lung-localized tissue-resident memory T cells developed at high numbers, circulatory cell migration was not required for their long-lasting persistence as a population in the lungs. These results are in line with prior reports that found that pre-existing lung-localized tissue-resident CD4 T cells were not affected by transient treatment with FTY720 (12, 20). Overall, this data suggests that inhibiting circulatory cell migration could be a viable treatment option for limiting allergic airway inflammation, but the effectiveness may be reduced in asthmatics that have long-term disease and have accumulated very high numbers of tissue-resident memory CD4 T cells.

## Discussion

Despite recent work that has explored the participation of tissue-resident memory CD4 T cells in allergic asthma, our knowledge about the contribution of allergen-activated circulatory cells in disease progression and in the maintenance of lung-localized Trm cells is very limited. Using an HDM-based murine model of periodic asthma exacerbations, our findings demonstrate that shortly after systemic priming of CD4 T cells, the migration of allergen-activated circulatory T cells is required for the generation, expansion, and long-lasting persistence of many Trm phenotype cells in the lungs. We also show that circulatory cell egress from the lymph nodes contributes to the effector memory T cell population that accumulates in the lungs and to the level of inflammation after Trm cells have formed. This suggests that migration of cells is critical to establishing the maximum tissue inflammatory activity in pre-sensitized allergic individuals. Lastly, we found that once allergen-induced Trm cells developed at high numbers, circulatory cell migration was not required for their persistence in the lungs. Overall, these data imply that future strategies might need to separately target existing Trm cells as well as circulatory memory T cells to fully inhibit CD4 T cell reactivity to airborne allergens and to fully limit exacerbations of asthma.

Our studies first showed that recurrent airway allergen exposures resulted in enhanced numbers of Trm phenotype cells accumulating in the lungs, which may represent both recently activated effector memory cells derived from circulating memory populations as well as those derived from existing resting Trm cells. A significant proportion (~75-80%) of these CD44^hi^CD62L^lo^CD69^+^ cells were protected from transient i.v. labeling, suggesting they were in the lung tissue. These findings are in line with previous reports also highlighting the development of Trm-like cells in the lungs of animals variably exposed to OVA and HDM (8–12). Similarly, our results with HDM confirm the long-term persistence of protected CD44^hi^CD62L^lo^CD69^+^ Trm phenotype cells in the lungs described previously with OVA as the antigen (12). Whereas the aforementioned studies largely focused on the net contribution of Trm cells to immediate recall activity to allergen, less attention was given to how the cells are formed and how circulatory/migratory T cells contribute to the Trm cell pool and to further exacerbations of lung inflammation. It is generally acknowledged that migration from secondary lymphoid organs to tissues is likely to be the first step in the initial formation of Trm cells. Interestingly, we observed the accumulation of HDM-reactive CD4 T cells in the lungs of systemically sensitized mice (**Supplementary Figure 2**) indicating that the allergen-activated circulatory CD4 T cells already started to migrate and accumulate in the lung tissues even before airway allergen exposure. Thus, these cells could be the foundation of some of the memory pool generated upon subsequent encounters with allergen, in addition to new cells that are recruited after allergen is inhaled. Corresponding to the latter, our studies with FTY720, which can block the egress of lymphocytes from lymphoid organs and thus prevents lymphocyte migration to inflammatory sites, confirmed that the initial development of the majority of allergen-induced lung Trm cells could be ablated. This was as long as FTY720 was given during both the initial secondary recall event to airborne allergen as well as the subsequent tertiary recall exposure to allergen. These results then suggest that inhibiting circulatory cell migration during the initial development of Trm cells could be a useful therapeutic strategy.

It was then of interest to determine if blocking migration of circulatory cells would either alter the maintenance of pre-existing lung Trm cell populations or the ability of animals to mount asthmatic inflammatory responses when such populations had already formed. Treatment with FTY720 during the tertiary recall response to allergen (**Figures 5A–D**) substantially suppressed lung inflammation, as indicated by reduced eosinophilia, peribronchial infiltrates, and mucus production, and resulted in a lower level of expansion of lung tissue-localized effector Trm phenotype cells. This shows that the relocation of circulatory cells into the lungs, even after Trm cells have developed, still contributes to exacerbation of asthmatic inflammation. Our data then suggest that targeting circulatory T cells during allergen insults might be employed to inhibit the accumulation of lung Trm cells in individuals with recent-onset asthma, and could be an excellent strategy to promote some form of airway tolerance. What would be the best method to inhibit the migration of circulatory memory T cells in asthmatics is not clear. Several S1P receptor modulators have been approved by the FDA for treatment of patients with multiple sclerosis (MS) or ulcerative colitis. However, their potential to be useful in asthmatics has not been fully evaluated. A small short-term phase II trial of FTY720 in patients with moderate asthma was performed to study possible safety issues with treatment of MS patients who are asthmatic (21). No primary issues were reported, although a separate case with one patient with a history of asthma with relapsing remitting MS noted a severe deterioration in lung function while on prolonged FTY720 treatment (22). Furthermore, a common adverse effect of S1P receptor modulators in patients is an increase in respiratory infections, and given the association of certain viruses with asthma exacerbations, such treatment would need to be given with caution. In this respect, potentially targeting integrins or similar adhesion molecules, or chemokines and their receptors, that are selectively needed for effector memory T cells to migrate into the lungs might be more appropriate.

Our work also highlights the possibility that treatment of asthmatics to reduce circulatory T cell migration might dampen acute inflammation and exacerbations of asthma even in long-term asthmatics, as long as the treatment was at the time of the exacerbation. However, the effectiveness is likely to be limited by the frequency of allergen-reactive Trm cells that have already accumulated in the individual’s lung tissue, and the relative contribution of these cells, versus effector memory T cells that come from the circulation, to recall allergen reactivity. This is based on the fact that in our study the long-lasting survival of the pool of pre-existing Trm cells did not depend on allergen-induced circulatory cells, with similar numbers of Trm cells persisting in the lungs at late times after allergen challenge regardless of FTY720 treatment (**Figures 5E, F**). Given that we show that repetitive exacerbations with allergen results in increasing numbers of Trm cells residing in the lungs, inflammation in more severe asthmatics that have a long history of exacerbations may be more strongly dependent on Trm cells as opposed to circulatory T cells. Thus, any benefit of modulating the migration of circulatory memory T cells could be limited in this case.

In summary, we provide more data to add to the growing literature on the importance of Trm cells to asthmatic lung inflammation, but also provide results to support a strong role for circulatory cells in contributing to recurrent exacerbations of asthma. Strategies that target the response of circulatory memory T cells and Trm cells together might be required to strongly inhibit T cell reactivity to airborne allergens and to limit exacerbations of asthma and their reoccurrence.

## Data availability statement

The original contributions presented in the study are included in the article/**Supplementary Material**. Further inquiries can be directed to the corresponding author.

## Ethics statement

This study was reviewed and approved by La Jolla Institute for Immunology Animal Care Committee.

## Author contributions

GS and DG conducted experiments. GS and MC designed experiments and wrote the paper. All authors contributed to the article and approved the submitted version.

## Funding

This study was funded by internal grants from the La Jolla Institute for Immunology to MC.

## Conflict of interest

The authors declare that the research was conducted in the absence of any commercial or financial relationships that could be construed as a potential conflict of interest.

## Publisher’s note

All claims expressed in this article are solely those of the authors and do not necessarily represent those of their affiliated organizations, or those of the publisher, the editors and the reviewers. Any product that may be evaluated in this article, or claim that may be made by its manufacturer, is not guaranteed or endorsed by the publisher.

## Supplementary material

Supplementary Figure 1
**(A)** Gating strategy to assess eosinophilia and CD4 T cells in BALF and lung tissue, and **(B)** memory/effector CD44hi CD4 T cells, and protected and non-protected CD44hiCD62LloCD69+ Trm phenotype cells, in lung tissue.

Supplementary Figure 2
**(A)** Experimental protocol to assess the effect of systemic sensitization on Th2 cytokine-producing CD4 T cells in the lung. Analysis on day 8. **(B)** Number of lung tissue CD44hi CD4 T cells. **(C)** Number of protected and non-protected CD44hiCD62LloCD69+ CD4 T cells. **(D)** Percentage of IL-5 and IL-13 producing lung CD4 T cells. Data means ± SEM, n = 4-5 mice per group, representative of 2 experiments.

Supplementary Figure 3 Summary of kinetic data from and . **(A)** Experimental protocol. **(B)** Number of lung tissue protected and non-protected CD44hiCD62LloCD69+ CD4 T cells over time. Bars depict the allergen exposure windows. Data means ± SEM, n = 4-8 mice per group, representative of 2 experiments.
